# *Lycium barbarum* polysaccharide attenuates high-fat diet-induced hepatic steatosis by up-regulating SIRT1 expression and deacetylase activity

**DOI:** 10.1038/srep36209

**Published:** 2016-11-08

**Authors:** Li Jia, Wang Li, Jianning Li, Yan Li, Hui Song, Yansong Luan, Hui Qi, Lirong Ma, Xiaohong Lu, Yi Yang

**Affiliations:** 1 Department of Biochemistry and Molecular Biology, Ningxia Medical University, Yinchuan 750004, China; 2 Institute of Endocrinology, Ningxia Medical University, Yinchuan 750004, China

## Abstract

In this study, we aimed to investigate the protective effects and underlying mechanism of *Lycium barbarum* polysaccharide (LBP) on high-fat-induced nonalcoholic fatty liver disease (NAFLD). Recently, sirtuin 1 (SIRT1) has been shown to play an important role in the regulation of hepatocellular lipid metabolism. Here, we demonstrated that LBP up-regulates SIRT1 deacetylase activity and protein expression by enhancing the NAD^+^/NADH ratio. Subsequently, LBP promoted LKB1 deacetylation and AMPK phosphorylation via SIRT1-dependent signalling. We also found that LBP increases acetyl-CoA carboxylase (ACC) phosphorylation and adipose triglyceride lipase (ATGL) protein expression and decreases fatty acid synthase (FAS) by activating the SIRT1/LKB1/AMPK pathway *in vitro* and *in vivo*. However, SIRT1 small interfering RNA (siRNA)-mediated knockdown reversed the LBP-mediated effects on ACC, FAS and ATGL. Moreover, LBP elevated carnitine palmitoyltransferase-1 alpha (CPT-1α) expression by suppressing malonyl-CoA accumulation. Taken together, our data indicate that LBP plays a vital role in the regulation of hepatic lipid metabolism and that pharmacological activation of SIRT1 by LBP may be a strategy for the prevention of NAFLD.

Increased dietary caloric intake causes obesity and subsequent inactivation of energy pathways, resulting in hepatic metabolic deterioration^1^. In this syndrome, the liver has an important role as the primary site for lipid deposition. High-fat-induced ectopic accumulation of triglycerides causes hepatocellular lipid metabolic derangements, contributing to increased nonalcoholic fatty liver and eventually to liver damage^1^,^2^,^3^. Therefore, efforts to elucidate the molecular mechanisms underlying lipid and energy metabolism are crucial for the development of new therapeutic strategies.

*Lycium barbarum* polysaccharide (LBP) is a novel antioxidant derived from wolfberry plants. Recent studies have suggested that LBP ameliorates dyslipidemia, promotes energy expenditure and reduces body weight, improving nonalcoholic steatohepatitis^4^,^5^. Our previous studies demonstrated that dysfunction of hepatic energy signalling induced by high-fat diet (HFD) represents a key mechanism for hepatic insulin resistance and lipid accumulation associated with nonalcoholic fatty liver disease (NAFLD). Additionally, treatment with LBP can lower systemic and hepatic triglycerides^6^. However, the underlying mechanism is not well understood. Our previous studies indicated that alpha-lipoic acid (ALA), a powerful antioxidant, ameliorates NAFLD and improves hepatic lipid metabolic syndrome by inducing the SIRT1/AMPK pathway *in vivo* and *in vitro*^7^,^8^. Recent studies suggested that defective SIRT1 in hepatocytes has a critical role of the development of NAFLD, as liver-specific *Sirt1*^*−/−*^ mice exhibit excessive accumulation of lipid droplets^9^,^10^,^11^. In addition, multiple reports demonstrated that there is a synergistic interaction between SIRT1 and AMPK in regulating lipid metabolism^8^,^9^,^12^,^13^,^14^. As we reported previously, hepatic SIRT1 improves lipid homeostasis by positively regulating AMPK to modulate lipid metabolic imbalance in response to long-term nutrient intake. AMP-activated protein kinase (AMPK) is an important regulator of energy metabolism and is directly activated by the intracellular AMP/ATP ratio^15^ or upstream liver kinase B1 (LKB1)^16^. Pharmacological activation of AMPK may be at least partially responsible for the therapeutic benefits on hyperlipidemia induced by long-term HFD.

Therefore, it is of interest to determine the potential benefits of LBP due to increases in SIRT1 or AMPK activity. We hypothesized that LBP may improve dysregulated lipid metabolism induced by HFD via SIRT1-dependent signalling. Here, we showed that LBP potently increases both SIRT1 deacetylase activity and AMPK phosphorylation activity, which in turn reduces triglyceride accumulation under high-fat conditions. This study provides novel mechanistic insight into NAFLD pathogenesis and indicates that LBP is a candidate for pharmacological intervention of NAFLD.

## Results

### LBP up-regulates SIRT1 deacetylase activity and protein expression by elevating the NAD^+^/NADH ratio

**Figure 1:**
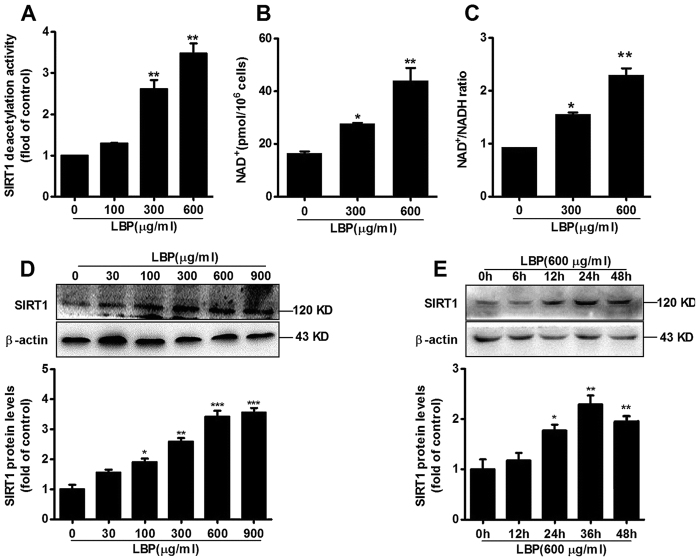
LBP activates SIRT1 deacetylase activity and protein expression by increasing the NAD^+^/NADH ratio. (**A**) The effect of different concentrations of LBP on SIRT1 deacetylase activity. Data are expressed as the mean ± SEM (n = 5). ^**^P < 0.01 *vs.* control. (**B**) Intracellular levels of NAD^+^ and (**C**) the NAD^+^/NADH ratio were measured in HepG2 cells incubated with LBP for 24 hours. Data are expressed as the mean ± SEM (n = 5). ^*^P < 0.05, ^**^P < 0.01 *vs.* control. (**D,E**) SIRT1 protein expression was determined by immunoblotting LBP-treated cells exposed to different concentrations or times. Data are expressed as the mean ± SEM (n = 3). ^*^P < 0.05, ^**^P < 0.01 ^***^P < 0.001 *vs.* control. All data were derived from three independent experiments.

To investigate the potential effects of LBP on SIRT1 activity and expression, we first examined SIRT1 deacetylase activity after exposure to LBP. Human SIRT1 recombinant protein and NAD^+^ substrate were coincubated with various concentrations of LBP for 1 hour. LBP substantially activated SIRT1 deacetylase activity in a dose-dependent manner (Fig. 1A). Along with the increase in SIRT1 activity, NAD^+^ levels and the NAD^+^/NADH ratio were significantly increased in LBP-treated HepG2 cells (Fig. 1B,C). Moreover, LBP significantly increased SIRT1 protein expression (Fig. 1D,E). Taken together, the results demonstrated that LBP up-regulates SIRT1 deacetylase activity and protein expression in a dose- or time-dependent manner.

### LBP promotes LKB1 deacetylation and AMPK phosphorylation

**Figure 2:**
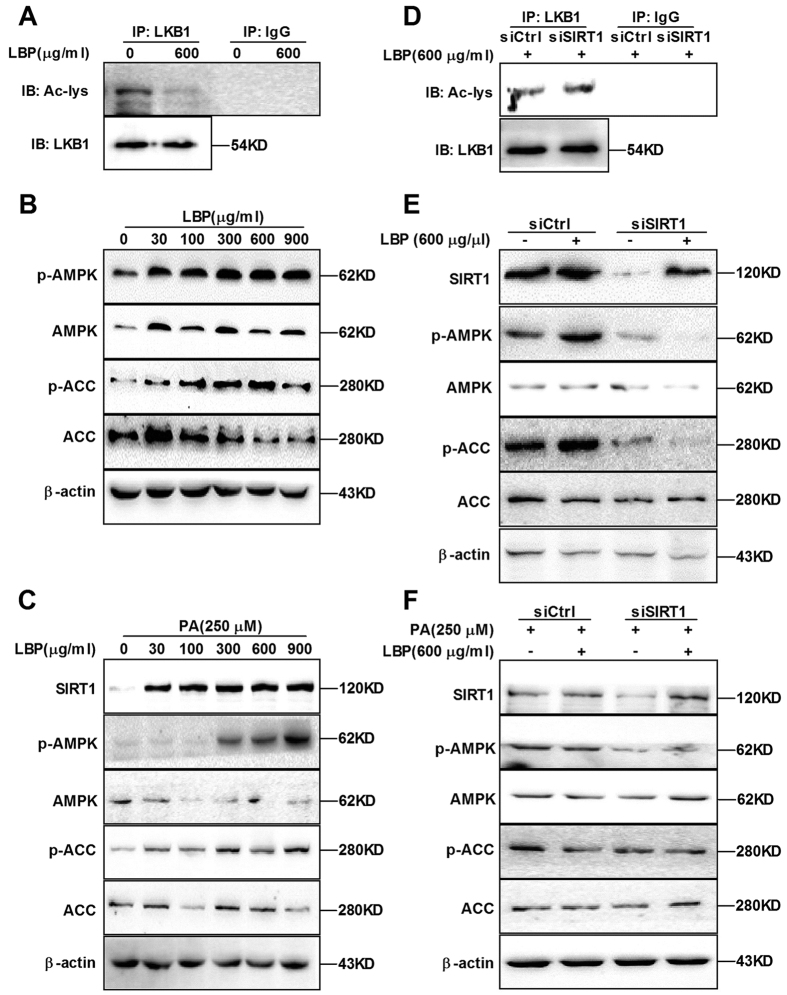
LBP increases LKB1 deacetylation and AMPK phosphorylation in a dose- or time-dependent manner. (**A**) Cells were treated with LBP for 24 hours and immunoprecipitated by a LKB1-specific antibody overnight. LKB1 acetylation was analysed by immunoblotting. (**B,C**) SIRT1, phospho-AMPK/AMPK and phospho-ACC/ACC protein expressions were examined by immunoblotting samples from LBP-treated cells in the absence or presence of PA. (**D**) Cells transfected by SIRT1 siRNA were treated with LBP for 24 hours. Cell lysates were immunoprecipitated by LKB1-specific antibody overnight, and the acetylation level of LKB1 was detected by immunoblotting. (**E,F**) SIRT1 and phospho-AMPK/ACC protein expressions were examined by immunoblotting in the absence or presence of PA. All results are representative of three independent experiments. *Note*: siCtrl, control siRNA; siSIRT1, SIRT1 siRNA.

AMPK phosphorylation is directly activated by LKB1; conversely, it is inhibited by LKB1 acetylation^8^,^9^,^16^,^17^. Thus, HepG2 cells were transfected with empty or SIRT1 plasmid for 48 hours and then incubated with anti-LKB1 overnight. As shown in Supplemental Fig. 1A, SIRT1 overexpression substantially reduced LKB1 acetylation. Furthermore, SIRT1 overexpression notably increased phosphorylation of AMPK and ACC in the absence or presence of palmitic acid (PA) (Supplemental Fig. 1B,C). Next we examined whether LBP promotes LKB1 deacetylation and AMPK phosphorylation. As expected, our results revealed that LBP not only elevated LKB1 deacetylation (Fig. 2A) but also increased phosphorylation of AMPK and ACC in the absence or presence of PA (Fig. 2B,C and Supplemental Fig. 2B). However, we found that SIRT1 siRNA knockdown led to a reduction in LBP-mediated LKB1 deacetylation (Fig. 2D). Additionally, LBP failed to reverse SIRT1 siRNA-decreased AMPK and ACC phosphorylation in the absence or presence of PA (Fig. 2E,F). These findings support the hypothesis that LBP promotes LKB1 deacetylation and AMPK phosphorylation in a SIRT1-dependent manner.

### LBP suppresses PA-induced hepatic triglyceride formation *in vitro*

**Figure 3:**
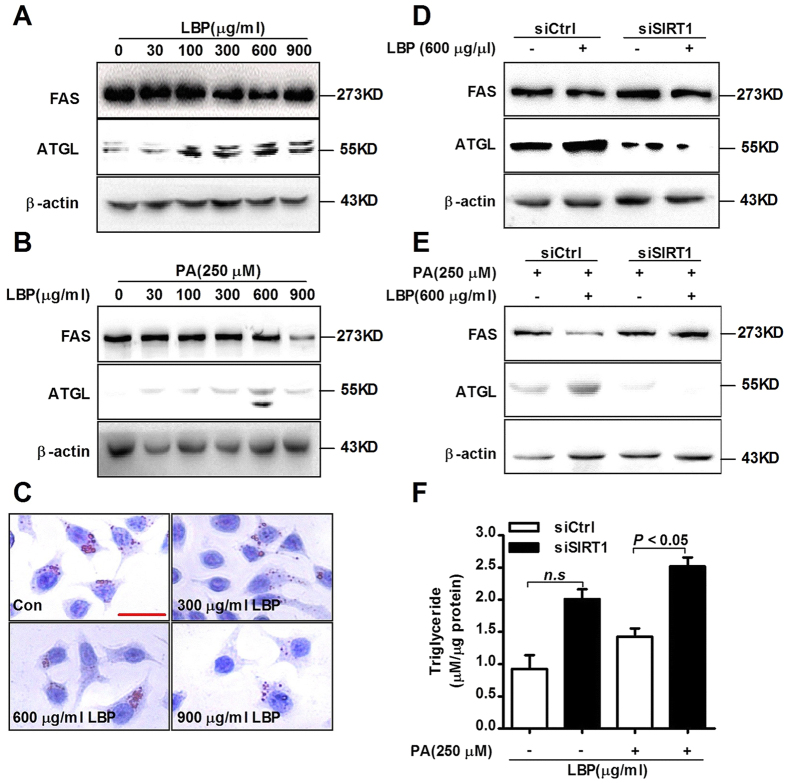
LBP significantly reduces hepatic triglyceride accumulation in cells chronically exposed to PA. (**A,B**) Cells were treated for 24 hours with different concentrations of LBP in the absence or presence of PA. ATGL and FAS protein expressions were examined by immunoblotting. All data shown are representative of three independent experiments. (**C**) Cells were treated with LBP and exposed to PA followed by Oil red O staining. (Scale bar: 7.5 μm). (**D,E**) Cells transfected by siRNA were treated with LBP in absence or presence of PA. All data shown are representative of three independent experiments. (**F**) After transfection by siRNA, cells were treated for 24 hours with LBP in the absence or presence of PA. Next, the triglyceride levels in each group were measured. Data are expressed as the mean ± SEM (n = 5). *Note*: *n.s*, non-significant.

To characterize the effects of SIRT1 in regulating hepatic lipid metabolism, cells were transfected with empty or SIRT1 plasmid for 48 hours and incubated with 250 μM PA for 12 hours. As showed in Supplemental Fig. 2A, SIRT1 overexpression suppressed FAS expression but promoted ATGL expression. To further determine whether LBP is involved in lipid metabolism, we examined downstream protein expression of the SIRT1/AMPK pathway. Our results suggested that LBP significantly reduced FAS expression in the absence or presence of PA, while it increased ATGL production (Fig. 3A,B). In addition, SIRT1 siRNA notably increased FAS expression and reduced ATGL expression in the absence or presence of PA (Fig. 3D,E). Conversely, pretreatment with LBP was unable to improve the above protein levels. Although SIRT1 expression of control siRNA-transfected cells was not changed in the absence or presence of LBP, the downstream phospho-AMPK, ATGL and FAS expressions were clearly altered (Figs 2E,F and 3D,E). Moreover, Oil red O staining indicated that LBP prevented PA-induced hepatic triglyceride formation (Fig. 3C and Supplemental Fig. 4). However, SIRT1 siRNA attenuated LBP-mediated hepatic triglyceride reduction (Fig. 3F). These results demonstrate that LBP suppresses PA-induced triglyceride accumulation by triggering the SIRT1/LKB1/AMPK pathway.

### LBP alleviates hepatic triglyceride accumulation in the HFD-induced NAFLD mice

**Figure 4:**
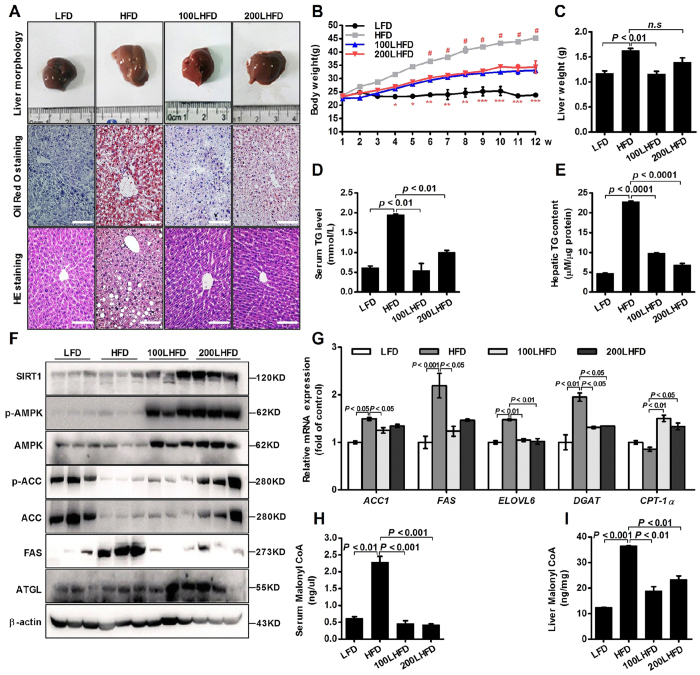
LBP significantly ameliorates hepatic steatosis in HFD-fed mice. (**A**) Representative liver tissue sections with Oil red O and H&E staining (scale bar: 15 μm). (**B**) Body weight changes and fresh liver weight in each group. Data are expressed as the mean ± SEM (n = 5). (**D,E**) Triglycerides were measured in each group. Data are expressed as the mean ± SEM (n = 5). (**F**) Immunoblotting analysis of SIRT1, phospho-AMPK/ACC, ATGL and FAS protein expressions. (**G**) The mRNA levels of lipid-related genes were measured by quantitative real time-PCR. Data are expressed as the mean ± SEM (n = 5). (**H,I**) Malonyl-CoA content was determined by an ELISA. Data are expressed as the mean ± SEM (n = 5). *Note*: LHFD, LBP + HFD.

To investigate the effects of LBP on lipid metabolism *in vivo*, we treated HFD-fed mice with a daily administration of LBP (100 and 200 mg/kg) for 12 weeks. Liver morphology, Oil red O and H&E staining showed that administration of LBP markedly alleviated hepatic triglyceride production and accumulation (Fig. 4A). At the beginning of 4 weeks, the body weight of HFD-fed mice showed a clear upward trend. From 6 weeks, the body weight of LBP-treatment mice was significantly decreased in the HFD-induced mice (Fig. 4B). Liver weights were decreased in the 200 LBP-treated mice (Fig. 4C). Additionally, LBP markedly reduced the triglyceride levels in the serum and liver (Fig. 4D,E). To further confirm the effects of LBP on the SIRT1/AMPK pathway *in vivo*, we examined SIRT1, phospho-AMPK, ACC, FAS and ATGL expressions. As shown in Fig. 4F, LBP significantly increased SIRT1, phospho-AMPK, ACC and ATGL expressions and decreased the FAS level in the HFD-fed mice. Moreover, LBP markedly decreased the mRNA expression of lipogenesis-related genes (*Acc1, Fas, Elovl 6* and *Dgat*), while it increased the mRNA expression of a lipolysis-related gene (*Cpt-1α*) (Fig. 4G). Previous studies reported that excess accumulation of malonyl-CoA can repress fatty acid oxidation via inhibition of CPT-1α activity^18^,^19^. Therefore, we investigated the effect of LBP on hepatocellular malonyl-CoA. As expected, our data showed that malonyl-CoA contents in the serum and liver were decreased in the LBP-treated mice (Fig. 4H,I).

### SIRT1 blocks hepatic lipogenesis in the HFD-induced NAFLD mice

**Figure 5:**
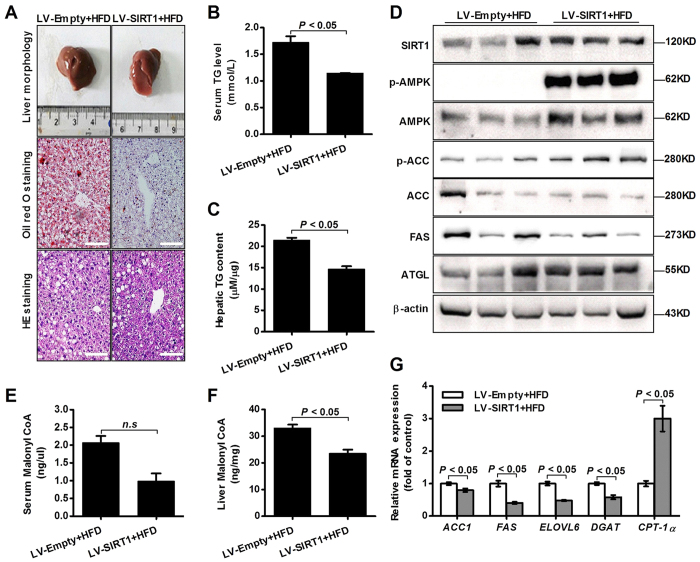
SIRT1 overexpression suppresses hepatic triglyceride accumulation in HFD-fed mice. (**A**) The liver sections of the LV-SIRT1 group were stained by Oil red O and H&E. (Scale bar: 15 μm). (**B,C**) Serum and hepatic triglycerides were measured. Data are shown as the mean+SEM (n = 5). (**D**) Immunoblotting analysis of the expression levels of SIRT1, phospho-AMPK/ACC, ATGL and FAS. (**E,F**) Malonyl-CoA level was determined by the ELISA. Data are expressed as the mean ± SEM (n = 5). (**G**) Quantitative real-time PCR analysis of the mRNA levels of lipid-related genes. Data are expressed as the mean ± SEM (n = 3).

To further confirm that SIRT1 regulated lipid metabolism, we injected HFD-fed mice with SIRT1 lentivirus (LV-SIRT1) via the tail vein for 12 weeks. As shown in Fig. 5A, liver section images indicated that LV-SIRT1 resulted in significantly decreased hepatic lipid accumulation in the HFD-induced NAFLD mice. The triglyceride content of the LV-SIRT1 mice was markedly reduced (Fig. 5B,C). We also examined the levels of downstream molecular components of the SIRT1-dependent pathway. The results showed that LV-SIRT1 substantially increased phospho-AMPK, ACC, and ATGL expressions and lowered FAS expression (Fig. 5D). In addition, LV-SIRT1 significantly increased mRNA levels of lipid-related genes (*Acc1*, *Fas*, *Elovl 6*, *Dgat* and *Cpt-1α*) (Fig. 5G). Moreover, our data suggested that LV-SIRT1 inhibited hepatic malonyl-CoA accumulation in the HFD-induced NAFLD mice (Fig. 5E,F).

### Loss of SIRT1 reverses the hepatic triglyceride reduction in the LBP-treated mice

**Figure 6:**
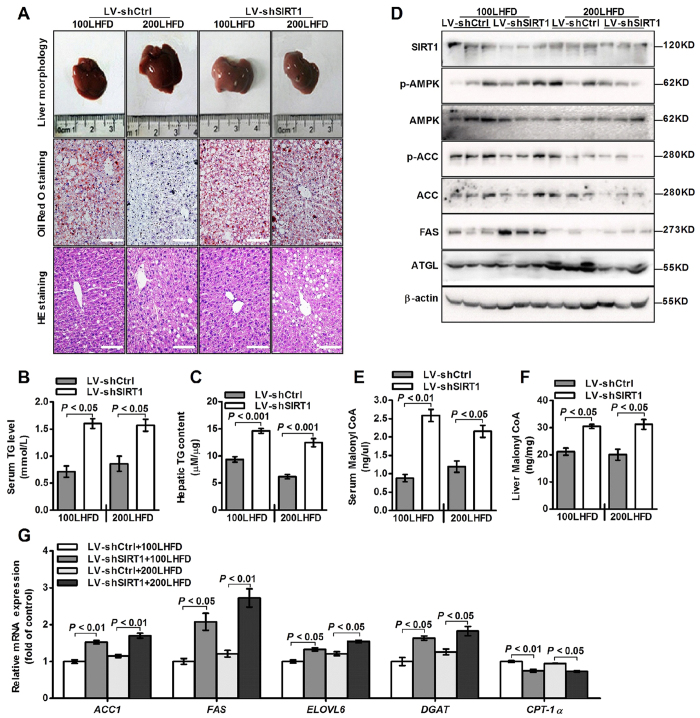
Knockdown of SIRT1 shRNA accelerates hepatic triglyceride accumulation in LBP-treated mice. (**A**) Representative images of mouse livers stained by H&E and Oil red O. (Scale bar: 15 μm). (**B,C**) Serum and hepatic triglyceride contents were measured. Data are shown as the mean + SEM (n = 5). (**D**) Immunoblotting analysis of SIRT1, phospho-AMPK/ACC, ATGL and FAS expressions. (**E,F**) Serum and hepatic malonyl-CoA levels. Data are expressed as the mean ± SEM (n = 5). (**G**) The mRNA levels of lipid-related genes were analysed by quantitative real time-PCR. Data are expressed as the mean ± SEM (n = 3).

To further elucidate the mechanisms underlying the anti-steatotic effect of LBP, we injected the SIRT1 shRNA lentivirus (LV-shSIRT1) via the tail vein of the LBP-treated mice at 12 weeks. As expected, morphological and histological images revealed that lipid droplets were markedly increased in the LV-shSIRT1 mice (Fig. 6A). Compared to the control shRNA lentivirus (LV-shCtrl) mice, triglyceride levels were substantially increased in the LV-shSIRT1 mice (Fig. 6B,C). LV-shSIRT1 reversed LBP-increased SIRT1, phospho-AMPK, ACC and ATGL expressions (Fig. 6D). Furthermore, LV-shSIRT1 inhibited LBP-reduced malonyl-CoA production (Fig. 6E,F). The mRNA expressions profiles were also reversed by LV-shSIRT1 (Fig. 6G). In summary, our studies showed that LBP can activate the SIRT1/AMPK pathway and ameliorate dysregulated hepatic lipid metabolism in HFD-induced NAFLD mice.

## Discussion

The liver is an important organ for lipid and energy metabolism, and the capacity of the liver to regulate lipid and energy metabolism is governed by a highly complicated regulatory network. Hepatic signalling regulation plays an important role in controlling hepatic lipid and energy metabolism in response to nutrient availability. Dysfunction of these signalling pathways is commonly linked to a number of high-fat diet-associated metabolic diseases, including type 2 diabetes, obesity and NAFLD. Increasing evidence indicates that SIRT1 systemically regulates lipid and energy homeostasis in many metabolic tissues by modulating a variety of signalling pathways^20^,^21^. For example, hepatic deletion of SIRT1 resulted in a significant increase in triglyceride accumulation in hepatocytes^11^,^22^, whereas SIRT1 overexpression significantly diminished triglyceride synthesis^23^,^24^.

**Figure 7:**
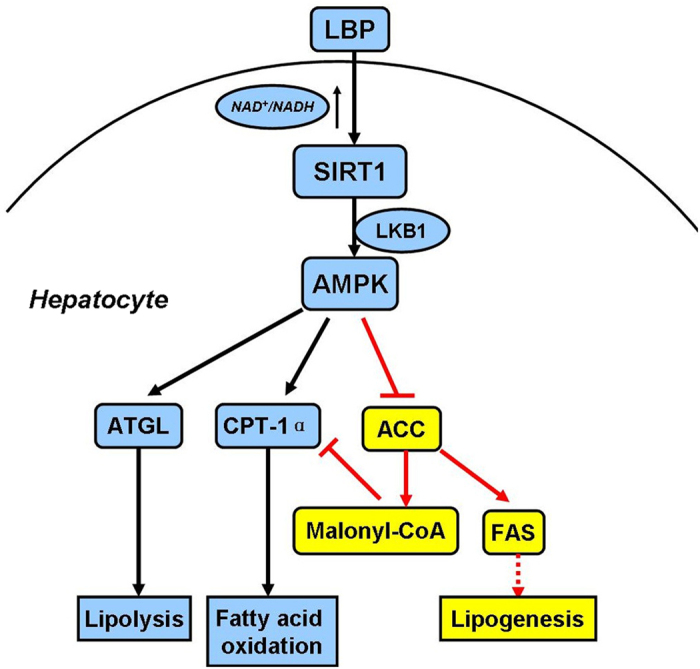
LBP regulates hepatic lipid metabolism under high-fat conditions by up-regulating the SIRT1/AMPK pathway.

Based on previous work by others and our laboratory, we hypothesized that LBP could be effective against NAFLD, dyslipidemia and metabolic syndrome. Given the important role of LBP in lipid metabolism signalling pathways, we first assessed the effect of LBP on intracellular SIRT1 deacetylase activity. As expected, LBP significantly up-regulated SIRT1 deacetylase activity and expression in a dose- or time-dependent manner *in vivo* and *in vitro* (Figs 1 and 4F). Several lines of evidence suggest a link between SIRT1 and AMPK, both of which are activated by food deprivation^11^,^25^ or pharmacological activation. For example, antidiabetic drugs, including metformin^26^ and ALA^8^,^9^, alleviate hepatosteatosis by activating the SIRT1 and AMPK signalling pathways. Here, we also showed that AMPK signalling was significantly impaired in SIRT1 siRNA-mediated knockdown models (Figs 2E,F and 6D), while increased SIRT1 expression stimulated AMPK phosphorylation (Figs 2B,C, 4F, 4D and Supplemental Fig. 1B,C). Recent studies found that LKB1, a key activator of AMPK^15^,^16^,^17^, is a direct substrate of SIRT1 deacetylase. Our data indicate that SIRT1 overexpression and LBP both significantly promoted the activation of AMPK through LKB1 deacetylation (Supplemental Figs 1A and 2A). However, knockdown of SIRT1 siRNA or SIRT1 shRNA lentivirus substantially reversed LBP-increased LKB1 deacetylation (Fig. 2D). This mechanism has been described for other SIRT1-mediated transcriptional activations, such as p53^27^, LXR^28^ and FoxO1^27^,^29^. In the present study, we identified SIRT1/AMPK as a central pathway affected by LBP. We observed that promoting the SIRT1/AMPK pathway by LBP results in increases in fatty acid oxidation and decreases in lipid formation (Fig. 7).

In addition, we demonstrated that LBP effectively improved hepatic triglyceride accumulation in HFD-fed mice and PA-treated cells (Figs 3C and 4A–E). The lipid-lowering effect of LBP was attributed to the increased ATGL and inhibited FAS protein production in hepatocytes (Fig. 3A,B, Supplemental Figs 2B and 4F). These responses were closely associated with SIRT1 deacetylase activity, showing that the lipid-related proteins were controlled by a SIRT1-dependent pathway (Supplemental Figs 2A, 5D and 6D). Our results were consistent with previous reports, which showed that activation of SIRT1 by pharmacological treatment has lipid-lowering effects through AMPK signalling^8^,^9^,^30^. Additionally, it is well known that AMPK phosphorylates and regulates several hepatic metabolic enzymes, such as ACC and FAS, which in turn increases fatty acid oxidation^31^. Our findings indicate that ACC activity was altered by LBP (Figs 2B,C, 4F and 5D and Supplemental Figs 1B,C and 2B). However, SIRT1 siRNA conversely increased ACC dephosphorylation *in vivo* and *in vitro* (Figs 2E,F and 6D). LBP inhibited lipogenic enzyme expression, e.g., *Acc1*, *Fas*, *Elovl 6* and *Dgat*, in HFD-fed mice (Figs 4G and 5G). These results demonstrated a novel mechanism by which LBP exerts a powerful lipid-lowering effect by activating the SIRT1/AMPK pathway and reducing lipid synthesis. Moreover, other researchers have shown that activation of ACC leads to increased intracellular malonyl-CoA, which blocks CPT-1α expression and abrogates fatty acid β-oxidation^32^. Surprisingly, LBP strongly upregulated CPT-1α expression and boosted fatty acid β-oxidation by repressing intracellular malonyl-CoA accumulation (Figs 4G–I and 5E–G). However, SIRT1-knockdown models further aggravated malonyl-CoA accumulation and suppressed CPT-1α mRNA expression *in vivo* (Fig. 6E–G).

Increasing evidence confirmed that LBP is involved in improving insulin sensitivity, and it has attracted attention as a novel antioxidant^33^. Originally, we explored the optimal incubation time of LBP-treated cells *in vitro*. We found that SIRT1 expression gradually increased in a time-dependent manner. However, after cells were incubated for 48 hours with LBP, SIRT1 expression was significantly reduced (Fig. 1E). These results were believed to be due to the fact that effective extracellular and intracellular LBP concentrations cannot be maintained over 24 hours. In addition, our data show that LBP significantly reduced body weight and liver weight (Fig. 4B,C). Moreover, Xiao, J. *et al*. reported that LBP improves hepatic functions in a cellular steatosis model^4^. Thus, we also used the palmitate acid-induced hepatocyte steatosis cell-based model and further explored the protective effect of LBP on fat-induced hepatocytes. Similarly, we found that LBP was able to inhibit the cellular steatosis model (Figs 3C and 4A). However, the effective molecular mechanism of LBP on high-fat-induced fatty liver is poorly understood. Thus, we found that the protective effects of LBP against steatosis were partly through the up-regulation of the SIRT1/AMPK pathway. In other words, we successfully showed that LBP prevents fatty liver induced by HFD; thus, it may potentially be used as a food supplement to prevent NAFLD.

In conclusion, the current study demonstrated a key role of LBP in attenuating high-fat-induced hepatic steatosis both *in vitro* and *in vivo*. The anti-steatotic effect of LBP was attributed to up-regulation of SIRT1 deacetylase activity and expression, revealing a new functional link between LBP and the SIRT1/AMPK pathway in hepatic lipid metabolism. Elucidating the role of LBP in hepatic steatosis may contribute to the development of a new therapeutic drug for NAFLD.

## Methods

### Ethics and method statement

The present experiments including animal subjects were approved by the Ethics Committee of Ningxia Medical University, Yinchuan, China. All methods were carried out in accordance with the approved guidelines.

### Reagents and materials

Foetal bovine serum (FBS) was obtained from the PAN (Germany) and HyClone. Cell culture materials were purchased from Corning Incorporated. Dulbecco’s modified Eagle’s medium (DMEM) was supplied by HyClone. Vigofect Reagent was purchased from Vigorous Biotechnology (Beijing, China). Lipofectamine 2000 was obtained from Invitrogen. Antibodies against phospho-AMPKα (#5831)/AMPKα (#2535), phospho-ACC (#3662)/ACC (#3661), LKB1 (#3050) and acetyl-lysine (#9441) were purchased from Cell Signaling Technologies. Antibodies against SIRT1 (13161-AP) and ATGL (55190–1-AP) were obtained from Proteintech. An antibody against FAS (ab22759) was purchased from Abcam. *Lycium barbarum* polysaccharide (LBP) was supplied by QiYuan Pharmaceutical of Ningxia Hui Autonomous Region (Yinchuan, China).

### Construction and transfection of SIRT1 overexpression vectors

The lentivirus overexpression vector (pCD315B-1) was purchased from System Biosciences (SBI). Full-length human SIRT1 was amplified using the following primers: forward: 5′-GCTCTAGCCACCATGGCGGACGA-3′; reverse; 5′-CGCGGATCCTGATTTGTTTGATGGATAGTT-3′. This ORF was inserted into the vector between the *Xba* I and *BamH*I sites. Empty or SIRT1 plasmid (5 μg) was transfected into cells separately with Vigofect Reagent according to the manufacturer’s instructions. After 48 hours of transfection, SIRT1 expression in the cells was assessed by immunoblotting. Recombinant lentiviruses encoding SIRT1 (LV-SIRT1) and a negative empty (LV-Empty) control were generated as described previously^34^.

### RNA interference design and transfection

To silence SIRT1, HepG2 cells were transfected with siRNA against human SIRT1 (33–100 nM) using Lipofectamine 2000 according to the manufacturer’s guidelines. The siRNA sequence was synthesized by Sangon Biotech (Shanghai, China) (SIRT1 siRNA: sense, 5′-UAGGUGCCCAGCUGAUGAAUU-3′, anti-sense, 5′-UUCAUCAGCUGGGCACCUAUU-3′; negative control siRNA: sense, 5′-UUCUCCGAACGUGUCACGUUU-3′, anti-sense, 5′-ACGUGACACGUUCGGAGAAUU-3′).The shRNA sequence (SIRT1 shRNA, 5′-GATCCTAGGTGCCCAGCTGATGAGTTCAAGAGACTCATCAGCTGGGCACCTATTTTTG-3′ or control shRNA, 5′-GATCCTTCTCCGAACGTGTCACGTTTCAAGAGAACGTGACACGTTCGGAGAATTTTTG-3′) was synthesized by Sangon Biotech and inserted into the pGreenPuro vector (SBI). Recombinant lentiviruses encoding mouse SIRT1 shRNA (LV-shSIRT1) or negative control shRNA (LV-shCtrl) were treated as described previously^34^.

### Animal experiments

Male C57BL/6 mice were purchased from the Animal Centre of Ningxia Medical University (Yinchuan, China). Mice were maintained in a standard cage on a 12/12 hour light-dark cycle. Six-week-old mice were fed a LFD containing 10% kcal from fat (MD12031, Medicience, China) or a HFD containing 60% kcal from fat (MD12033, Medicience, China) for 12 weeks. HFD-fed mice were also pretreated with 100 or 200 mg/kg LBP for 12 weeks. For overexpression of SIRT1 in the liver, 1.0 × 10^8^ plaque-forming units (PFU) of LV-SIRT1 or LV-Empty were injected into HFD-fed mice via the tail vein in accordance with approved guidelines as previously described^35^. To knock down SIRT1 in the liver, 1.0 × 10^8^ PFU LV-shSIRT1 or LV-shCtrl was injected into LBP-treated mice. At week 13, mice were fasted for 8 hours and sacrificed for experimental analysis. All animal procedures were conducted according to the protocols approved by the Committee of Animal Use for Research and Teaching of Ningxia Medical University, Yinchuan, China (document number: SCXK (ning) 2015–0001).

### Cell culture and treatments

HepG2 cells (Academy of Medical Sciences Tumour Cell Libraries, Beijing, China) were grown in DMEM supplemented with 10% FBS and 1% (v/v) penicillin-streptomycin at 37 °C and 5% CO_2_. HepG2 cells were frequently cultured for 24 hours, followed by treatment with various concentrations of LBP (30–900 μg/mL) for 24 hours and 25 μM PA for 12 hours.

### Measurement of SIRT1 activity

SIRT1 deacetylase activity was measured using a SIRT1 Fluorometric Drug Discovery Kit (Enzo Life Sciences) according to manufacturer’s instruction.

### NAD^+^/NADH ratio assay

The NAD^+^/NADH ratio of whole-cell extracts of HepG2 cells was measured using a NAD^+^/NADH Quantification Colorimetric Kit (BioVision) according to the manufacturer’s instructions.

### Immunoprecipitation and immunoblotting

Immunoprecipitation assays were performed as previously described^7^,^8^. Proteins (100–200 μg) from cell lysates were incubated with anti-LKB1 overnight and then incubated with Protein A/G beads (Santa Cruz Biotechnology) at 4 °C for 4 hours. Immunoprecipitates were washed with cold PBS and boiled with sample loading buffer for 5 min. Immunoprecipitates and sample proteins (50 μg) were separated by 10% SDS-PAGE and transferred to polyvinylidenedifluoride (PVDF) membranes (Millipore). Membranes were blocked with 5% nonfat milk at room temperature for 1 hour followed by incubation with primary and secondary antibodies. Membranes were developed with an enhanced chemiluminescence system (Thermo Scientific) and detected under Azure Biosystems (USA).

### Determination of triglyceride content

Triglyceride content was measured using a Triglyceride Assay Kit (Applygen Technologies, Beijing, China) according to the manufacturer’s protocols.

### Quantitative real-time PCR

Total RNA was extracted from the liver tissues using TRIzol reagent (Invitrogen) and was transcribed to cDNA using the Superscipt First Strand Synthesis Kit (Thermo Scientific) in accordance with the manufacturer’s protocols. Real-time PCR analysis was performed using KAPA SYBR Kit on *qTOWER* 2.0. The primer sequences are listed in the Supplemental Table 1. Real-time PCR reactions were performed in triplicate and normalized to β-actin.

### Malonyl-CoA content measurement

Malonyl-CoA contents in the serum and liver tissues were determined using an ELISA Kit according to the manufacturer’s instructions (CUSABIO).

### Statistical analysis

All results are expressed as the mean ± SEM. Data between groups were analysed by Student’s *t*-test or one-way ANOVA. Differences between groups were considered statistically significant at *P* < 0.05.

## Additional Information

**How to cite this article**: Jia, L. *et al*. *Lycium barbarum* polysaccharide attenuates high-fat diet-induced hepatic steatosis by up-regulating SIRT1 expression and deacetylase activity. *Sci. Rep.* **6**, 36209; doi: 10.1038/srep36209 (2016).

**Publisher’s note:** Springer Nature remains neutral with regard to jurisdictional claims in published maps and institutional affiliations.

## Supplementary Material

Supplementary Information
