# Supplementary material for: Lycium barbarum polysaccharide attenuates high-fat diet-induced hepatic steatosis by up-regulating SIRT1 expression and deacetylase activity
Source: Sci Rep. 2016 Nov 8;6:36209. doi: 10.1038/srep36209 (PMC5099939; doi:10.1038/srep36209)
Supplement: Supplementary Information [file srep36209-s1.doc]

**Supplementary Information**

***Lycium barbarum* polysaccharide attenuates high-fat diet-induced hepatic steatosis by up-regulating SIRT1 expression and deacetylase activity**

Li Jia ^1, 2, *^, Wang Li ^1, 2, *^, Jianning Li ^1, 2^, Yan Li ^1, 2^ , Hui Song ^2^, Yansong Luan ^1, 2^, Hui Qi ^1, 2^, Lirong Ma ^1, 2^, Xiaohong Lu ^1, 2^ and Yi Yang ^1, 2^

^1^Department of Biochemistry and Molecular Biology, Ningxia Medical University, Yinchuan 750004, China.

^2^Institute of Endocrinology, Ningxia Medical University, Yinchuan 750004, China.

^*^These authors contributed equally to this work. Correspondence and requests for materials should be addressed to Y.Y. (e-mail: [yangyi73422@163.com](mailto:yangyi73422@163.com)).

**Methods**

***Lycium barbarum* polysaccharides (LBPs) information.** *Lycium barbarum* (*L. barbarum*) berries have been used in the China as a traditional medicinal herb and food supplement. *L. barbarum* polysaccharides (LBPs) are the primary active components of *L. barbarum* berries and possess a wide array of pharmacological activities, which is thought to be mainly due to its high LBPs content. *L. barbarum* berries contain abundant LBPs (including six main monosaccharaides present: arabinose, D-(+)-glucose, galactose, mannose, L-(-)-fucose and xylose) and chemical ingredients. LBPs are considered the most important functional constituents in *L. barbarum* berries ^1^.

**Quantitative analysis of Oil red O staining.**

HepG2 cells were incubated for 12 hours using 250 μM PA, and then treated for 24 hours with varying doses of LBP (100, 300, 600 and 900 μg/ml). Cells were washed with cold PBS and fixed with 4% paraformaldehyde for 10 min at room temperature, and then stained with Oil red O for 15 min. Cells were then washed twice with 60% isopropanol and dissolved in 100% isopropanol, and then quantified by spectrophotometrical analysis at 600 nm ^2^.

**MTT assay.**

Cell viability was evaluated by the MTT assay. After LBP-treatment, cells from each group were washed by cold sterile PBS and then incubated with 5 mg/ml MTT (Sigma-Aldrich) for 3 hours, and subsequently dissolved in dimethyl sulfoxide (DMSO). The absorbance of cells was measured at 570 nm.

**Supplemental Table 1. Gene-specific primer sequences for real-time PCR ana**lysis

| Gene | Forward primer | Reverse primer |
| --- | --- | --- |
| *ACC1* | AGCAGATCCGCAGCTTG | ACCTCTGCTCGCTGAGTGC |
| *FAS* | TTCCAAGACGAAAATGATGC | AATTGTGGGATCAGGAGAGC |
| *ELOVL6* | ACAATGGACCTGTCAGCAAA | GTACCAGTGCAGGAAGATCAGT |
| *DGAT* | TTCCGCCTCTGGGCATT | AGAATCGGCCCACAATCCA |
| *CPT-α* | TCTTGCAGTCGACTCACCTT | TCCACAGGACACATAGTCAGG |
| *β-actin* | GGAAATCGTGCGTGACATTA | TCAGGCAGCTCGTAGCTCTT |

**Supplemental Figures Legends**


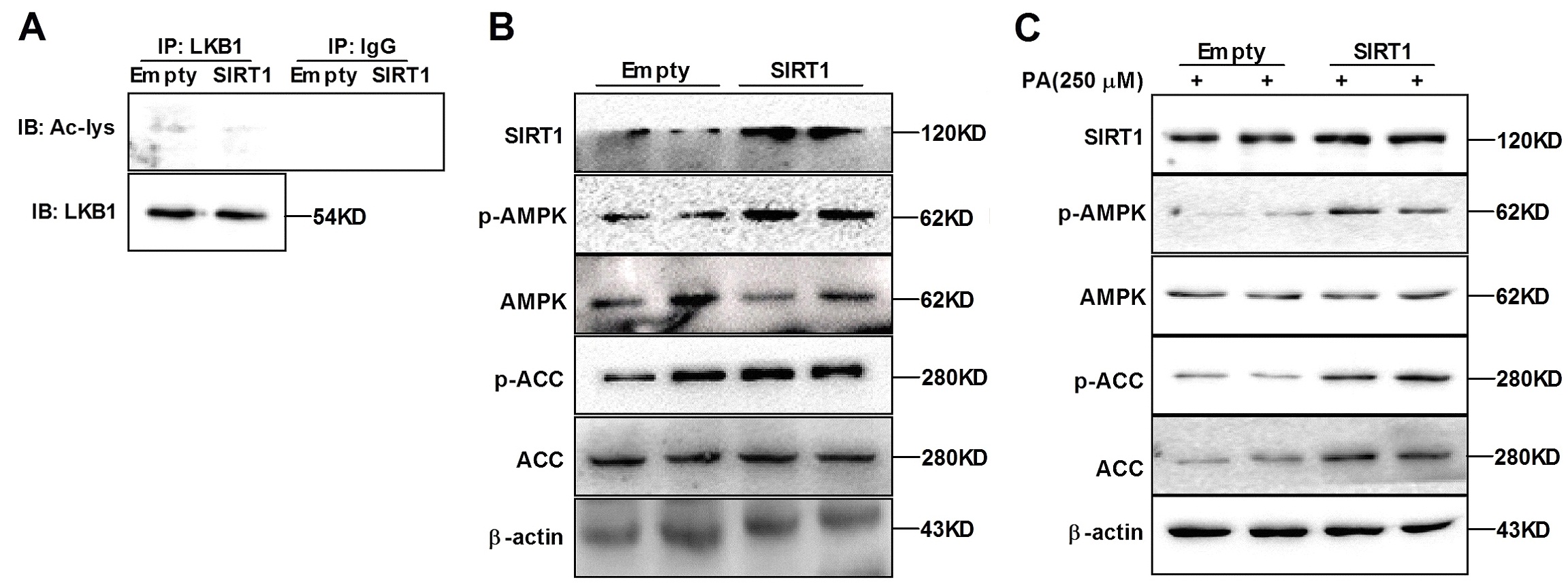


**Supplemental Fig. 1︱LKB deacetylation and AMPK phosphorylation is regulated by SIRT1-dependent pathway.** (A) Cells were transfected with empty or SIRT1 plasmid for 48 hours, and immunoprecipitated by anti-LKB1 for overnight. IgG-specific antibody was normalized to the control. (B, C) Immunoblotting analysis of SIRT1, phospho-AMPK/ACC proteins expression in SIRT1-overexpressed cells the absence or presence of PA. All results shown are representative of three independent experiments.


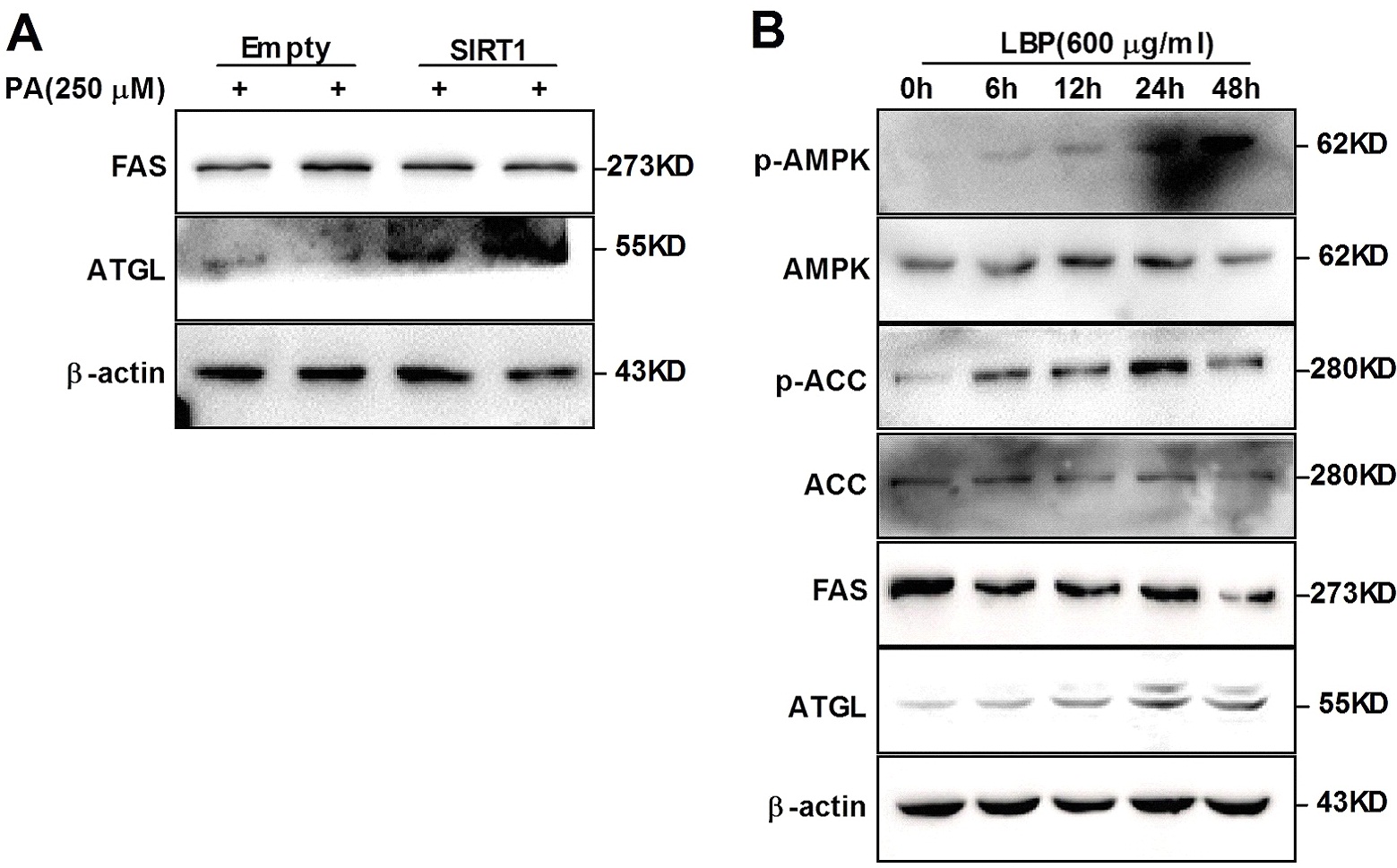


**Supplemental Fig. 2︱Effects of LBP on the downstream molecules expression of SIRT1/AMPK pathway.** (A) Cells were transfected with empty or SIRT1 plasmid in the presence of PA. (B) Cells were pretreated with LBP at different time. ATGL and FAS protein expression were analyzed by immunoblotting. All results shown are representative of three independent experiments.


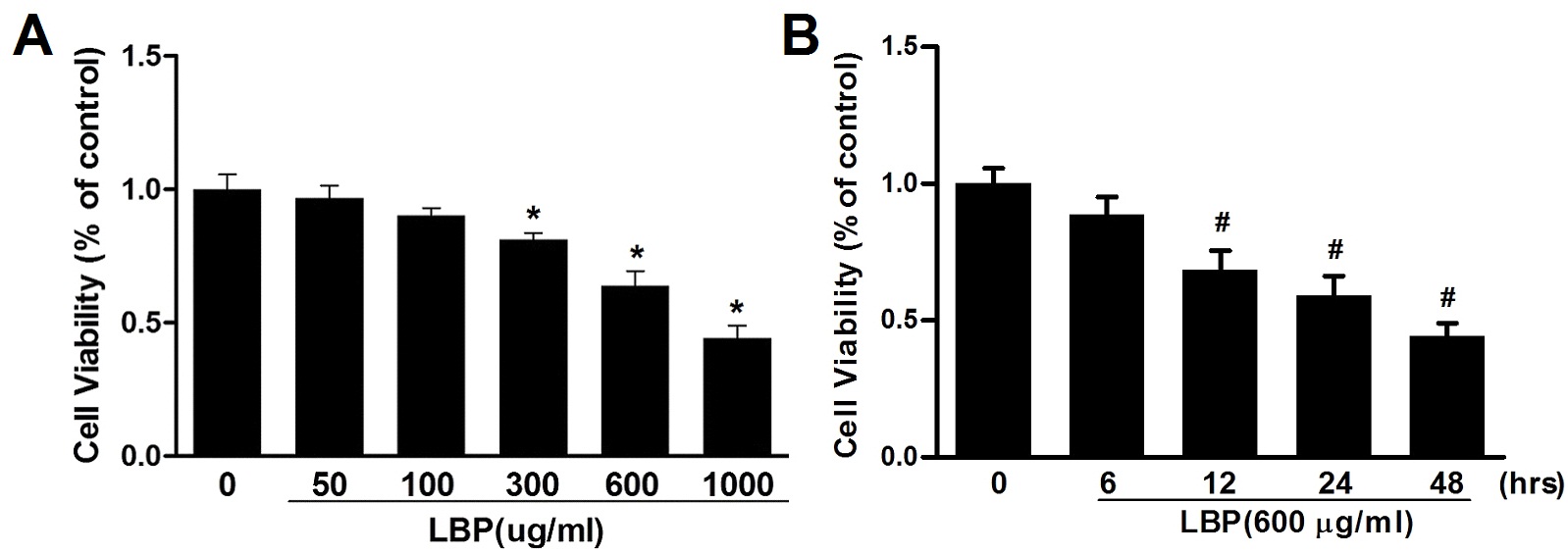


**Supplemental Fig. 3︱MTT experiment analysis of LBP toxicity in the cells.** (A) Cells were treated for 24 hours with different concentration of LBP. (B) Cells were treated with LBP at different time.


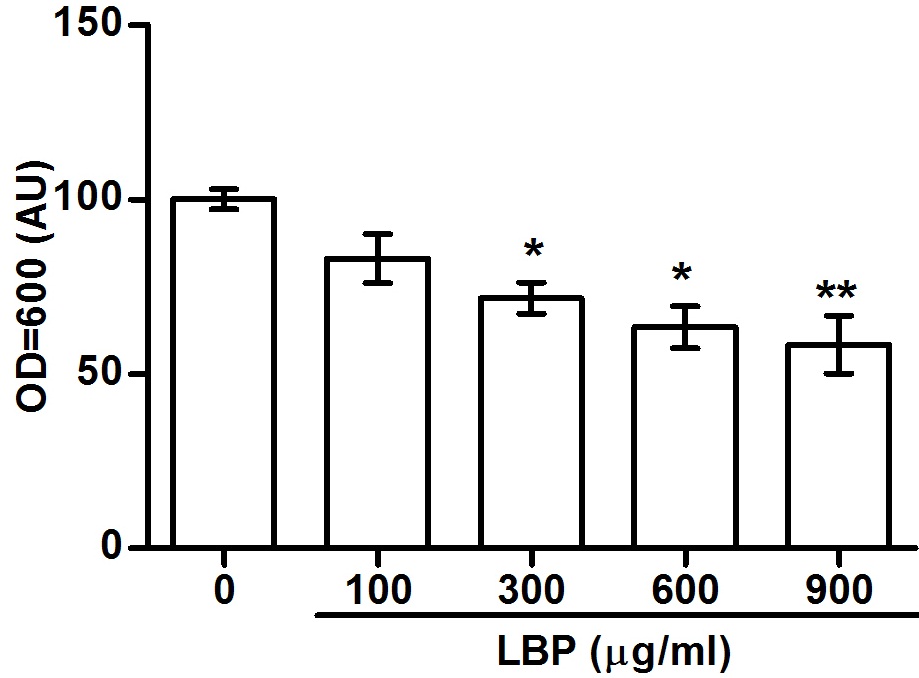


**Supplemental Fig. 4︱**Cells were incubated for 12 hours using PA and treated with LBP for 24 hours. The absorbance of cells stained Oil red O was measured at 600 nm.

**References**

1. Cheng, J. *et al*. An evidence-based update on the pharmacological activities and possible molecular targets of lycium barbarum polysaccharides. *Drug Design, Development and Therapy*. **9**, 33-78 (2015).

2. Soomin, C., Youngshim, C., Soyoung, P. & Taesun, P. Carvacrol prevents diet-induced obesity by modulating gene expressions involved in adipogenesis and inflammation in mice fed with high-fat diet. *Journal of Nutritional Biochemistry*. **23**, 192-201 (2011).
